# Global report on preterm birth and stillbirth (5 of 7): advocacy barriers and opportunities

**DOI:** 10.1186/1471-2393-10-S1-S5

**Published:** 2010-02-23

**Authors:** Megan Sather, Anne-Véronique Fajon, Rachel Zaentz, Craig E Rubens, the GAPPS Review Group

**Affiliations:** 1 GMMB, 1010 Wisconsin Ave, NW, Suite 800, Washington, DC 20007, USA; 2 Global Alliance to Prevent Prematurity and Stillbirth, an initiative of Seattle Children's, Seattle, Washington, USA; 3 Department of Pediatrics at University of Washington School of Medicine, Seattle, Washington, USA

## Abstract

**Background:**

Efforts to achieve the Millennium Development Goals (MDGs) to improve maternal and child health can be accelerated by addressing preterm birth and stillbirth. However, most global health stakeholders are unaware of the inextricable connections of these adverse pregnancy outcomes to maternal, newborn and child health (MNCH). Improved visibility of preterm births and stillbirths will help fuel investments and strengthen commitments in the discovery, development and delivery of low-cost solutions globally. This article addresses potential barriers and opportunities to increasing global awareness and understanding.

**Methods:**

Qualitative research was conducted to analyze current knowledge, attitudes and commitments toward preterm birth and stillbirth; identify advocacy challenges; and learn more about examples of programs that successfully advocate for research and appropriate interventions. Forty-one individuals from 14 countries on six continents were interviewed. They included maternal, newborn, and child health advocates and implementers, United Nations agency representatives, policymakers, researchers, and private and government donors.

**Results:**

A common recognition of three advocacy challenges with regard to preterm birth and stillbirth emerged from these interviews: (1) lack of data about the magnitude and impact; (2) lack of awareness and understanding; and (3) lack of low-cost, effective and scalable interventions. Participants also identified advocacy opportunities. The first of these opportunities involves linking preterm birth and stillbirth to the MDGs, adding these outcomes to broader global health discussions and advocacy efforts, and presenting a united voice among advocates in the context of broader MNCH issues when addressing preterm birth and stillbirth. Another key opportunity is putting a human face to these tragedies—such as a parent who can speak to the personal impact on the family. Lastly, several interviewees suggested identifying and engaging champions to garner additional visibility and strengthen efforts. Ideal champions will work collaboratively with these and other maternal, newborn and child health issues. Conclusion: Advocacy efforts to add preterm births and stillbirths to broader MNCH goals, such as the MDGs, and to identify champions for these issues, will accelerate interdisciplinary efforts to reduce these adverse outcomes. The next article in this report presents an overview of related ethical considerations.

## Background

"Solid advocacy needs to be based on solid evidence, and it is therefore unacceptable that the scientific activity and the political awareness and action in this field are low" 

Andres de Francisco, M.D., Ph.D., M.Sc., D.T.M&H 

Partnership for Maternal, Newborn and Child Health

Although preterm birth and stillbirth are two specific global health problems among the broader maternal, newborn and child health (MNCH) issues, they have not been elevated to the same level on the MNCH agenda in terms of resources, funding and media attention. As discussed in article 1 on data [1], more than one million babies die as a result of complications surrounding pre-term birth—it is a leading cause of neonatal death and increases risks for many short- and long-term health problems [2]. Additionally, more than 3.2 million stillbirths occur each year [3].

Preterm birth and stillbirth are nearly invisible to the majority of health decision makers globally. Yet, both issues are of growing concern among health experts, scientists, and advocates in low- and middle-income countries (LMICs) and in high-income countries (HICs). Deaths due to preterm birth are on the rise in many HICs—despite having health systems with more resources to devote to improving maternal, newborn and child health outcomes [4].

Despite the magnitude of these public health burdens, many causes of both preterm birth and stillbirth remain largely unknown. While research has been undertaken in HICs, no effective interventions have been identified to prevent preterm birth. Additionally, there is a lack of systematic evaluations of solutions to reduce the burdens of both outcomes, particularly in LMICs. Few believe enough resources have been allocated to thoroughly research these issues.

Raising awareness and advocating for these issues has been difficult because of the lack of knowledge about the magnitude of the health, economic, and social impacts of preterm birth and stillbirth. The lack of low-cost, scalable interventions with proven impact for low-resource settings remains a barrier to advocating for more resources and funding.

The interviews conducted for this article provide insight into current attitudes and commitments of global health leaders and organizations, financial partners, researchers, healthcare practitioners and other key stakeholders. This will enable advocates to examine the best strategies to implement successful, sustained advocacy efforts to prevent preterm birth and stillbirth.

### Focus of article

This article summarizes qualitative research on advocacy issues relating to global preterm birth and stillbirth. The research sought to answer the following questions:

• What is the extent of knowledge on preterm birth and stillbirth?

• Who has made these issues priorities, and why?

• What has prevented governments and organizations from making them a priority?

• What would drive future commitment and focus?

• What are the advocacy elements of successful programs working on these issues?

• What are the main challenges to conducting preterm birth and stillbirth advocacy?

Information on attitudes, behaviors, value systems, concerns, motivations, aspirations, cultures and lifestyles was gathered through phone and e-mail interviews. This article is not intended to be used as a comprehensive scientific accounting of all relevant advocacy issues, and should not serve as an advocacy plan for organizations focusing on preterm birth or stillbirth. Rather, this article highlights issues and raises questions to inform the work of global stakeholders. The analysis and article do not reflect the opinions or recommendations of the interviewers.

## Methods

To conduct a balanced and comprehensive landscape review, interviews were conducted with a multidisciplinary sampling of representatives from a diverse number of organizations around the globe. Forty-one individuals working on issues related to preterm birth and stillbirth participated in the research interviews, including maternal, newborn and child health (MNCH) advocates and implementers; representatives from United Nations (UN) agencies; Non-Governmental Organizations (NGOs); policymakers; academics; researchers; and private and government donors.

**Figure 1:**
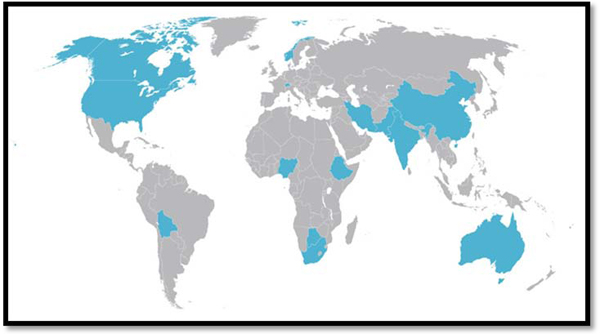
Geographic span of research interviews.

Together, the interviewees, located in 14 countries across six continents, offered a global perspective to the advocacy landscape of preterm birth and stillbirth (Figure 1).

Interviews were conducted by phone and e-mail. A complete list of interviewees is provided in Additional file 1.

An interview guide was developed to facilitate conversations. Using this guide, each interview was tailored to address the specific experience and perspective of the interview subject. Throughout the course of the project, the researchers continually updated the guide to reflect current events and emerging issues. The average interview was approximately 50 minutes long.

This article is based on the interviews as well as follow-up conversations and materials provided by the interviewees.

## Results

There is a perception that little research has been conducted about preventing preterm birth and stillbirth. The result is a critical knowledge gap among various audiences about the causes of preterm birth and stillbirth, as well as successful interventions and their potential impact. Consequently, it is challenging to secure financial and policy support that leads to higher awareness and visibility, a unified policy community, successful partnerships, consistent messaging, increased funding, and, ultimately, reduction of these major global health crises.

### Challenges to advocacy

#### Lack of knowledge about the magnitude and impact of preterm birth and stillbirth

This first section explores the three most frequently referenced challenges to a successful advocacy program:

• The lack of evidence-based knowledge (data) about the magnitude and impact of preterm birth and stillbirth

• The lack of awareness and understanding of preterm birth and stillbirth

• The lack of low-cost, effective prevention methods and interventions for preterm birth and stillbirth 

"I think the biggest thing that's going to drive commitment and focus is to have…solid data on the magnitude of the problem. supported by doable interventions that are currently available…and showing the potential impact [of] those simple interventions"

Mark Young, M.D., MHSc

United Nations Children's Fund (UNICEF)

#### The problem of data on preterm birth and stillbirth

Country-level data on the prevalence and causes of preterm birth and stillbirth are largely unavailable and inadequate. Interviewees agree the lack of data greatly impedes efforts to get preterm birth and stillbirth on the global health agenda:

"…if this information was consistently there every time you talk about maternal or infant mortality then the issue would get the visibility and the attention that it needs"

Nils Daulaire, M.D., M.P.H.

Global Health Council

"…a lack of awareness and education about these deaths has hampered prevention efforts. In addition, there is a need for better data collection to help identify ways to prevent these deaths"

Senator Frank R. Lautenberg, D-NJ

United States Senate

Interview participants reported the following problems with data collection:

• Preterm births and stillbirths are largely under-reported—whether in HICs or LMICs.

• In most LMICs, health systems do not have the infrastructure and capacity to support the reporting of health data, especially on preterm birth and stillbirth.

• Many women in rural settings do not deliver in facilities or with skilled attendants—if a baby is pre-term or stillborn, no formal record is kept.

• There is often stigma associated with stillbirths, so women and families may keep them secret. As such:

"…many stillbirths [and preterm births] go undetected and don't even become part of the statistics'.'

Scott Jackson, M.B.A., C.F.R.E.

PATH

Interviewees also identified important challenges in collecting reliable data in HICs.

• Because there are no national or international standards for reporting preterm births or stillbirths or for registries themselves, different information—and different levels of information—is recorded in different places, creating an inadequate and inconsistent database of information.

• In order to create an international standard for reporting preterm births and stillbirths, international global health and government leaders must come to a consensus on some of the questions posed by some of our interviewees:

- What are the rights of a stillborn?

- Should stillbirths receive a birth certificate, a death certificate, or neither?

- When a preterm baby or stillbirth is delivered, what is the standard set of data about the mother's background and health that should be recorded?

- Are there any environmental factors that should be recorded?

Interviewees broadly agreed that the lack of data—such as statistics, causes, and benchmark reports—is the main reason that countries, regardless of geographic location and GDP, have not made preterm birth and stillbirth priorities. The lack of data leaves important questions unanswered for policymakers and funders:

• What is the magnitude of these issues?

• What are the causes of these issues?

• What can we do about these issues?

• What are the costs and cost savings associated with addressing these issues?

One interviewee reflected on her government: 

"I think that principally our government hasn't done much because they don't know the magnitude of the problems, and if they know the magnitude, they don't know what to do"

Bertha Pooley, M.P.H.

Save the Children, Bolivia

Another interview participant noted that advocates in this area can learn from similar efforts, particularly around neonatal deaths, where there have been similar challenges in lack of routine data collection and social taboos in mourning. The issue of newborn health is rising in importance on the global health agenda—not necessarily because of more data, but because of convincing systematic global estimates of numbers and causes of death, clearly framed links to the MDGs and a strong consensus on doable interventions:

"[The issues of stillbirth and preterm birth] do not have to wait decades for perfect data—improved estimates and clear communication of the relevance of these for existing national goals and program investment is possible and crucial"

Joy Lawn, BMedSci, M.B. B.S., M.R.C.P. (Paeds), M.P.H.

Save the Children, Saving Newborn Lives

#### No existing metrics on the economic and societal impacts of preterm birth and stillbirth

While estimates of the magnitude of preterm birth and stillbirth do exist, these estimates are not the reliable numbers that advocates need in order to obtain funding for programmatic change. However, there are other ways to measure the impact, especially the economic and social impacts, of preterm birth and stillbirth. The results of which could be effectively used to raise awareness of the issues and advocate for resources or funding.

Interview participants noted that the lack of information on the economic burden of preterm birth and stillbirth—a burden shared by governments, businesses and families—is debilitating, and that costing studies would be a very useful tool for advocates approaching policymakers and funders. Interviewees referenced the economic burden associated with the following list:

• Preterm birth

- Delivering and keeping preterm babies alive

- The potential long-term health consequences of preterm babies (health, education, etc.)

- Women opting out of the workforce to take care of babies with health problems as a result of preterm birth

- Additional cost of closer scrutiny to future pregnancies after a preterm birth

• Stillbirth

- Lost work days of mothers and fathers grieving for their stillbirths

- Mental health and grief counseling

- Women no longer participating in the workforce due to complications with pregnancy that can be avoided with prenatal care

- Additional cost of closer scrutiny to future pregnancies after the delivery of a stillbirth

Multiple experts indicated that because the majority of policymakers are men, hard numbers resonate more with topics like maternal health, where it might be harder to relate to personal stories about the plight and emotional burden of preterm birth and stillbirth for women and families. As Jill Sheffield stated about advocating to the G8 for maternal health funding:

"The people who make decisions about the [allocation] of resources need [to know about] the economic impact [those resources and funds will have on the problem]."

Jill Sheffield

Family Care International

Interviewees also reported the social and emotional impact that preterm birth and stillbirth have on families and communities:

• Preterm birth

- The emotional and financial strain that can be put on a family by the birth and the subsequent health conditions of a preterm baby

- The integration issues a preterm child, enduring physical or learning disabilities, may face later in life

• Stillbirth

- The psychological and emotional burden on families having experienced stillbirth

- The repercussions that having grief-stricken, anxious parents can have on siblings of a stillbirth.

Because a stillbirth does not result in a baby needing care, multiple interviewees suggested that the public health burden of stillbirth is difficult for some policymakers and funders to understand—and therefore to allocate resources. As such, it will be important to explore and cost out the social impact of stillbirth to illustrate the public health burden of this issue.

The lack of knowledge about preterm birth and stillbirth—promulgated by absent data and assessments of their economic and societal impact—has meant few opportunities for advocates to raise awareness of these issues.

#### Lack of awareness and understanding of preterm birth and stillbirth

##### Limited awareness of the impact of preterm birth and stillbirth 

There are varying opinions on the degree to which different audiences—the healthcare community, policymakers, donors, the general public, and others—are aware of preterm birth and stillbirth as major health issues. However, there is general consensus that awareness and knowledge are sparse and incomplete, suggesting a long and important road ahead for advocacy and awareness-raising efforts.

United States-based physicians and researchers reported a significant amount of research being conducted on preterm birth and stillbirth, and that these issues are priorities for obstetrician-gynecologists, neonatologists and pediatricians in HICs. According to these interviewees:

• In HICs preterm birth receives far more attention than stillbirth, even within the medical community, as it is increasingly viewed as a public health burden with economic repercussions.

• While there are studies on the causes of preterm birth, none have yielded conclusive findings to advance the prevention of preterm birth. Preterm birth rates continue to rise in both HICs and LMICs (Uma Reddy, M.D., M.P.H., NICHD).

• Though researchers have studied several therapies that could be used to prevent preterm birth, progesterone remains the only promising intervention at this time. (Uma Reddy, M.D., M.P.H., NICHD)

Others reported the following:

• The general public typically becomes aware of preterm birth and stillbirth through personal stories: giving birth to—or having an acquaintance deliver—a preterm baby or a stillborn.

• Few audiences, including the medical community in HICs and LMICs, fully understand the link between preterm birth and all of the associated long-term health consequences.

- Though people acknowledge the frailty of a preterm baby and potential complications that may ensue, they do not always establish linkages between preterm birth and potential repercussions such as cerebral palsy, vision problems, learning disabilities and others.

• There exists a discrepancy in levels of awareness between HICs and LMICs.

- Preterm birth and stillbirth are better known and recognized in countries like the United States, for example, where the medical community receives continuous training and organizations like the March of Dimes and First Candle conduct public education campaigns and political advocacy.

- Preterm birth and stillbirth are relatively unknown in LMICs where preterm births and stillbirths are unreported, and few countries have stillbirth reporting systems.

##### Cultural nuances and perceptions surrounding preterm birth and stillbirth

In LMICs, long-standing cultural misconceptions concerning preterm birth and stillbirth have perpetuated false information and beliefs, especially at the community level. Based on the experience of health campaigns and other similar advocacy efforts, these cultural beliefs, varying from country to country, will have to be factored into any successful advocacy plan (Theresa Shaver, M.P.H., R.N.M., White Ribbon Alliance).

Interviewees identified numerous cultural nuances and perceptions about preterm birth and stillbirth. Below are some that could present challenges to advocacy efforts:

• Misconceptions about the prevention of preterm birth and stillbirth

- Preterm birth and stillbirth are inevitable— experts referred specifically to the deep sense of fatalism, hopelessness and defeat felt in many LMIC societies. In fact, in certain countries, babies are not named until they have survived their first year, given the high rates of neonatal mortality. In one interviewee's words, there is often a sense that:

"… this is the way it's always been and it's the way it always will be"

Nils Daulaire, M.D., M.P.H.

Global Health Council

- Stillbirth is sometimes seen as nature's way of intervening—when a baby is stillborn, some believe this means it was not meant to be born at that time, or had a physical or mental malformation. (Pang Runyan, M.D., M.P.H, Peking University).

• Societal treatment of preterm birth and stillbirth

- Stillbirths are kept secret and are not to be discussed or mourned publically—families in LMICs (and in many HICs as well) view stillbirth as a taboo subject.

"Stillbirths are hidden. We know very little. It's kept quiet"

Vinod Paul, M.D., Ph.D., F.A.M.S., F.I.A.P., F.N.N.F.

All India Institute of Medical Sciences

"…in many cultural situations there is a general mindset not to discuss stillbirth in an open fashion."

George Little, M.D.

Dartmouth Medical School

- Preterm birth or stillbirth is perceived as the mother's fault, which leads to feelings of shame and remorse for women.

•  Misconceptions related to science, medicine, and development

- Stillbirths are like miscarriages—some believe that stillbirths do not have the same standing as babies who are born alive.

- Some health officials in LMICs believe that preterm babies and low birth weight babies are the same. While most HICs differentiate gestational age from birth weight, this is not the case yet for many in international health circles, pointing to a need for knowledge and education so that preterm birth rates can be reported accurately, and the right interventions can be sought for both preterm birth and stillbirth.

- In certain countries preterm babies are abandoned—especially girls—when the costs to treat medical problems that result from a preterm birth are worth more than the baby's life itself to the family. Also, families in some countries assume preterm babies will always be weak and do not know that with proper care, preterm babies can thrive.

- When preterm babies survive a number of days or reach certain milestones, they are perceived to be "in the clear" without future medical needs. This notion is often perpetuated by news stories of miracle babies and by physicians failing to mention the long-term consequences a preterm baby may endure (Simin Taavoni, M.Sc., Iran University of Medical Sciences). Stillbirth is too unbearable for some organizations (e.g., private sector partners) to associate with, and preterm birth is an easier cause to advocate for because it still results in a baby that needs ongoing treatment and assistance, and not in a death.

There is also a perception that there are little or no consequences on a baby's health and development if the delivery is scheduled several weeks early via C-section for the mother's convenience—a trend that is growing rapidly in HICs.

##### Tailored approach needed for successful advocacy

As the examples illustrate, interview participants firmly believe that no country is the same when it comes to cultural nuances and perceptions surrounding maternal health, childbearing, newborn health, and other related issues. For successful advocacy, cultural nuances and perceptions must be closely examined and efforts must be tailored and adapted for each country and implemented by actors who will be cognizant of the cultural sensitivities at play (Femi Kuti, M.B.B.S, F.R.C.O.G., Obafemi Awolowo University, Nigeria). As one participant stated, this will be a matter of examining:

"…the major issues for each country.rather than [taking] a global perspective."

Marian Sokol, Ph.D., M.P.H.

First Candle

To dispel some of the false cultural notions that are associated with preterm birth and stillbirth, interviewees suggest that advocates will need to build strategies based on reliable global, regional, and local data that are not available at this time.

The lack of knowledge about the magnitude and impact of preterm birth and stillbirth, and the ensuing lack of awareness and understanding globally, remain some of the largest obstacles to conducting successful advocacy efforts. The advocacy challenge that must be resolved is not only quantifying the magnitude and the impact of preterm birth and stillbirth, but also presenting low-cost, effective interventions:

"…showing what potential impact those simple interventions could have on the problem [. ] that's the most powerful thing that's needed."

Brian Hansford

United Nations Children's Fund (UNICEF)

#### Lack of simple, cost-effective interventions for preterm birth and stillbirth

"If they can't propose coherent solutions, then policymakers are going to move on to the next issue. They'd rather put their resources into something they know they can do something about."

Jeremy Schiffman, Ph.D.

Syracuse University

Assessing the impact and magnitude of preterm birth and stillbirth is an important piece of the puzzle in creating a successful advocacy roadmap, though as many pointed out, it cannot be the whole puzzle:

"…For governments and organizations to make this a priority, […] providing them with some examples of what can actually be done is going to make a huge difference"

O. Massee Bateman, M.D., D.T.M&H.

Save the Children

In order to achieve this, one of the most important goals for preterm birth and stillbirth advocates will be to research and identify interventions that meet four key criteria:

• Low-cost

• Effective

• Scalable

• High impact

Interviewees agreed that as of yet, there is no single and straightforward intervention or package of interventions for preterm birth and stillbirth that meet all of these criteria. As one interviewee acknowledged, policymakers and funders may recognize that preterm birth and stillbirth are important issues, but they do not have a clear, low-cost and scalable solution comparable to those of diarrheal disease, pneumonia and diseases requiring antibiotics or vaccines (Bertha Pooley, M.P.H., Save the Children Bolivia). While the effectiveness of Kangaroo Mother Care was referenced by multiple interview participants, several also noted that it is not an intervention that can be used in isolation.

Multiple interviewees reported that preterm birth and stillbirth are indicators for maternal health, and that maternal health, in turn, can be considered an indicator for the strength, effectiveness and capacity of a country's health system. A high number of preterm births and stillbirths in LMICs—many of the latter happening at birth—could be prevented if women had better access to regular care and quality prenatal care, and had skilled attendants or access to a health facility at delivery in the event of a labor complication. There is a growing consensus that a high number of preterm births resulting in newborn deaths could be prevented by home and community-based newborn care that includes preventing or treating neonatal infections (Abhay Bang, MD, MPH, Director, SEARCH).

These interventions, while taken for granted in the Western world, are not simple and low-cost in LMICs, where they have major infrastructure implications: education, training, technology, and transportation, among others (Ana Langer, M.D., Engender Health and Bertha Pooley, M.P.H., Save the Children, Bolivia).

Several interviewees indicated that an increased emphasis on preventive interventions for preterm birth and stillbirth may resonate with policymakers and funders (Vinod Paul, M.D., Ph.D., F.A.M.S., F.I.A.P., F.N.N.F., All India Institute of Medical Sciences). A change in focus is needed to shift from saving preterm newborns to preventing preterm births and reduce the strain on countries' health systems:

"If you increase the number of [preterm newborns] and reduce mortality, you still have not guaranteed good health to mothers and children, because once a child is preterm there is no guarantee that they will be able to actually catch up and grow up to be as robust as a child that was carried full-term"

Joy Phumaphi

World Bank

According to many interviewees, successful advocacy outreach efforts to policymakers, thought leaders, and funders will need to provide a defined roadmap for action, or clear steps to creating that roadmap (Rachel Wilson, M.P.H., PATH). Steps may include demonstrating the magnitude of these issues, the impact they have on societies and country budgets, and how allocating funds to developing and scaling low-cost, scalable interventions can provide an important return on investment. Advocacy should include efforts to encourage donors, the United Nations, and other organizations to incorporate the scale-up of priority interventions in their funding strategies. In other words, as one participant succinctly explained:

"…showing them it can be done. It can be done, and it doesn't [have to] cost much"

Monir Islam, M.D., M.P.H.

Making Pregnancy Safer, WHO

### Opportunities for advocacy

#### Developing a new framework to position preterm birth and stillbirth

The following section presents the advocacy opportunities identified by interview participants, including:

• Linking preterm birth and stillbirth to the United Nations Millennium Development Goals (MDGs)

• Adding preterm birth and stillbirth to broader global health discussions

• Presenting a united voice

• Putting a human face on the issues

• Identifying and engaging champions

"I think when you can put a face [and] a name [to] a real story and present that to public policy makers, suddenly they get it and that's one of the huge […] opportunities that we have now'.'

Marian Sokol, Ph.D., M.P.H.

First Candle

Interview participants across sectors identified several advocacy opportunities for advocates, including GAPPS, its partners, and like-minded organizations working to place preterm birth and stillbirth on the global health agenda. In addition, participants from Save the Children, UNICEF, March of Dimes and White Ribbon Alliance shared best practices from their experience conducting successful, high-visibility MNCH advocacy campaigns.

#### Linking preterm birth and stillbirth to the Millennium Development Goals (MDGs)

Without addressing preterm birth and stillbirth, MDG 4 will not be achieved, as preterm birth is a primary cause of neonatal death. In addition, investing in interventions to prevent stillbirth would align with and support the reduction of maternal mortality and neonatal death, thereby addressing both MDGs 4 and 5. As one participant expressed, the prevention of preterm birth and stillbirth has not yet been recognized as crucial to achieving the MDGs (Andres de Francisco, M.D., Ph.D., M.Sc., D.T.M&H, Partnership for Maternal Newborn and Child Health). This said, the halfway point to 2015 has passed, and policymakers, funders, and thought leaders across the globe are focused on crossing the finish line while assessing what is achievable, and what is not. As one participant said:

"…governments, at this point in time, are focused on Millennium Development Goal 4 and 5"

Zulfiqar Bhutta, M.B.B.S., F.R.C.P., F.R.C.P.C.H., F.C.P.S., Ph.D.

Aga Khan University, Pakistan

Another participant explained that donors who fund MNCH programs tend to focus either more on child health (e.g., United States, Canada) or maternal health (e.g., Netherlands, Sweden, UK), leaving room for advocates to link preterm birth and stillbirth to MDGs 4 and 5 and promote a continuum of care (Helga Fogstad, M.H.A., NORAD).

With regard to stillbirth in particular, a participant noted that there is enormous ground to be gained by linking stillbirth to maternal health outcomes:

"…advocates might consider linking preterm birth advocacy to infant and child health, and linking stillbirth explicitly to maternal health, as stillbirth is often assumed to be an indicator of poor maternal health"

Ann Starrs, M.P.A.

Family Care International

A question for preterm birth and stillbirth advocates is:

"How do you create a framework that links […] preterm birth and stillbirth to overall child survival goals, so that by having a campaign dedicated to preterm birth and stillbirth, you are adding to the conversation about the funding, the public awareness of the broader maternal and child health MDGs and goals that people have had some focus on."

Scott Jackson, M.B.A., C.F.R.E.

PATH

This framework presents advocates with a unique opportunity to advocate for resources and funding, within the context of achieving MDGs 4 and 5, aimed at preventing preterm birth and stillbirth.

#### Adding preterm birth and stillbirth to larger global health discussions

The majority of interview participants for this research do not view preterm birth and stillbirth as "stand-alone" issues. Rather, each is intimately nestled into a larger MNCH framework (though neither receives enough visibility). Several interviewees offered that, in order to conduct the most successful advocacy effort, the existing MNCH community should be mobilized:

"I would recommend that this group gets seen as an additive…to be able to create a whole set of new materials that can help other people who are already advocating for the bigger piece of the pie. To have additional stories, additional messages, additional champions to add into the pot as opposed to carving out their own part that might take away from others…"

Rachel Wilson, M.P.H.

PATH

One participant suggested that, in order to be successful, advocates would need to develop a phased approach to advocacy, one that focuses first on raising awareness of the scale and impact of preterm birth and stillbirth. With more awareness, advocacy efforts could shift to winning political support and funding to continue to identify interventions and solutions. (Ann Starrs, M.P.A., Family Care International)

#### Presenting a united voice for preterm birth and stillbirth

Along these same lines, interview participants identified the need to bring together different sectors working across the many aspects of MNCH to have a unified voice on preterm birth and stillbirth. For example, Occupational Safety and Hazard offices—which exist in many countries and are perhaps not obvious partners at first glance—can play an important role in LMICs by regulating work conditions to limit adverse outcomes for mothers (Naomi Cassirer, Ph.D., International Labour Organization).

Or, in many countries, funds for MNCH programming are held by multiple entities: both Ministries of Health and Ministries of Finance, but also ministries dealing with women and family, nutrition, or infrastructure. There is an opportunity to convene these allies from across many different sectors and advocate in a united voice.

There is also an opportunity for organizations working on different aspects of preterm birth and stillbirth— research, advocacy and implementation, for example—to convene and learn about efforts and initiatives being carried out and commit to working as a more cohesive, multi-disciplinary entity. When asked who they believed are the leaders on preterm birth and stillbirth, interviewees most frequently identified organizations in their own fields or within their own countries and regions. This said, some marked trends appeared, including:

• WHO and UNICEF were frequently recognized as leading authorities for global data and statistics

• The International Stillbirth Alliance was often referred to by interviewees as the first body to have started an global conversation about stillbirth

• First Candle, March of Dimes, Save the Children, and White Ribbon Alliance were the most frequently named organizations for advocacy activities

There is a visible opportunity for organizations across spanning sectors and disciplines to convene and synchronize efforts to and elevate the issues of preterm birth and stillbirth in a united voice.

#### Illuminating the human face on the issues

While illustrations of the global economic and social impact of preterm birth and stillbirth have the potential to resonate loudly with funders and policymakers, the sharing of personal stories can also be a powerful and effective approach to raising awareness on these issues, especially stillbirth. The best advocates for resources to prevent stillbirth can be the parents who have experienced this tragedy and can speak to its impact on their lives. U.S. Representative Peter King, J.D. (R-NY) was so moved when a constituent shared her story with him that he introduced the Stillbirth Awareness and Research Act of 2008.

"Her story and the story of so many other women and their families are by far the most effective way to show legislators that more action is necessary, especially at the federal level"

Peter King, J.D., R-NY

United States House of Representatives

Advocates like Marian Sokol of First Candle, Vicki Flenady of the International Stillbirth Alliance, and others confirm that parent advocates all over the world have been on the front lines of stillbirth advocacy efforts, and have achieved successes thanks to their efforts.

"Parent groups like SIDS and Kids were drivers with the SIDS agenda; […] the wonderful work they've done in this country in reducing SIDS has laid a very strong foundation for the stillbirth initiative"

Vicki Flenady, M.Med.Sc.

Centre for Clinical Studies, the Mater Mother's Hospital, Australia

#### Identifying and engaging champions

Several interview participants commented on the important role that champions can play in garnering visibility for an issue. One interviewee specifically referenced political leaders, first ladies, queens, actors, and women who have had a personal experience as potentially powerful ambassadors for the issues of preterm birth and stillbirth (Ana Langer, M.D., Engender Health).

Multiple participants referenced Sarah Brown's work as a Global Patron of the White Ribbon Alliance as a model case study. Cultivating a relationship with a champion for preterm birth and stillbirth and building a successful campaign around their stories may provide an excellent opportunity for advocates to effectively reach media, policymakers, funders, the medical community and the general public to elevate these issues.

With an eye on 2015, there is a lot of activity and momentum in the global health and the MNCH community and advocates have an unprecedented opportunity to complement and strengthen existing efforts.

### Issues for consideration

While there is a range of opinion among the interview participants about the knowledge and awareness of the impact of preterm birth and stillbirth, there is general consensus that raising awareness and advocating for these issues has been difficult and has been an obstacle to the development of scalable, cost-effective interventions. To that end, several key questions were raised throughout the interviews, and several are flagged here for consideration:

• The challenge of creating an advocacy and messaging framework for preterm birth and stillbirth within the existing MNCH and MDG frameworks: messaging for MNCH and the MDGs are already established. A smart strategy is needed to create a framework for preterm birth and stillbirth within these two existing frameworks so as not to compete, but rather, add to the conversation.

• Consistency of messaging around preterm birth: Because of the complexity of the issue and potential interventions, existing preterm birth advocacy messages can be at cross purposes: currently, one set of messages focuses on provision of proper care to preterm newborns so that they can thrive after birth; another highlights prevention in order to avoid a lifetime of health and societal challenges that are costly and consume vast resources to address.

• The importance of prioritizing interventions: Interview participants agreed that there is no consensus on which interventions should be used, especially in LICs. Furthermore, most agree that there needs to be a shift toward more preventive interventions but that this has not yet been addressed by the global health community in a constructive way.

• The need to come to consensus on numbers: The research, medical, global health and NGO communities must come to agreement on the preterm birth and stillbirth estimates to use in messaging and advocacy materials. Without consistent numbers, it will be extremely difficult to convey a clear and powerful message to policymakers and funders.

## Conclusion

According to interview participants across sectors and in different areas of the world, there is broad acknowledgement of significant scientific, policy and advocacy knowledge gaps on the issues of preterm birth and stillbirth. Interviews with a diverse sampling of representatives from the nonprofit sector, non-governmental organizations, UN agencies, global health organizations and others revealed three major challenges to successful advocacy efforts on the issues of preterm birth and stillbirth:

• A lack of knowledge of the magnitude and impact of preterm birth and stillbirth

• A lack of understanding and awareness of the issues

• A lack of effective, low-cost, scalable and highly impactful interventions that take into consideration the existing cultural beliefs within each setting

There are, however, opportunities that exist in advocating to prevent preterm birth and stillbirth—notably an opportune timeframe with the MDGs as governments focus on achievable results; a mobilized maternal, newborn and child health community; and parent advocates who are succeeding in putting a face on the public health tragedies. Interview participants broadly acknowledge that the global stage is set for advocacy on preterm birth and stillbirth, though many questions—some difficult— remain to be addressed.

Ethical and social justice considerations relating to preterm birth and stillbirth are discussed in the next article of this report [5]. Preceding articles in this report discuss data, discovery science, existing interventions and delivery strategies [1,6-8]. The final article presents a collaborative global action agenda created by leading stakeholders [9].

## Authors' contributions

The article was written and reviewed by MS, AF and RZ. CR helped conceive of this article as part of a global report on preterm birth and stillbirth, and participated in its design and coordination.

## Competing interests

The authors declare that they have no competing interests.

## Supplementary Material

Additional File 1
