# Supplementary material for: Global report on preterm birth and stillbirth (5 of 7): advocacy barriers and opportunities
Source: BMC Pregnancy Childbirth. 2010 Feb 23;10(Suppl 1):S5. doi: 10.1186/1471-2393-10-S1-S5 (PMC2841773; doi:10.1186/1471-2393-10-S1-S5)
Supplement: Additional File 1 [file 1471-2393-10-S1-S5-S1.pdf]

Additional File 1

Interview Participants

Organization/Affiliation	Name	Title	Country
**Aga Khan University, Department of Pediatrics & Child Health**<br><br>**GAPPS**	Zulfiqar Bhutta, M.B. B.S., F.R.C.P., F.R.C.P.C.H., F.C.P.S., Ph.D	Husein Lalji Dewraj Professor, Chairman<br><br>Core Investigator	Pakistan
**All India Institute of Medical Sciences**	Vinod K. Paul, M.D., Ph.D., F.A.M.S., F.I.A.P., F.N.N.F.	Professor of Pediatrics, Head of the Division of Neonatology;<br>Director of the WHO Collaborating Centre for Training and Research in Newborn Care	India
**Bill & Melinda Gates Foundation**<br><br>**GAPPS**	Gary Darmstadt, M.D.	Senior Program Officer for Neonatal Health in Integrated Solutions Development, Global Health Program<br><br>SAC Member	USA
**Burroughs Wellcome Fund**	John Burris, Ph.D.	President	USA
**Burroughs Wellcome Fund**	Rolly Simpson	Administrator	USA
**Centers for Disease Control and Prevention (CDC)**<br><br>**GAPPS**	Eve Lackritz, M.D.	Chief of the Maternal and Infant Health Branch, Division of Reproductive Health<br><br>SAC Member	USA
**Dartmouth Medical School**	George Little, M.D.	Professor and Neonatologist	USA
**Engender Health**	Ana Langer, M.D.	President and CEO	USA
**Family Care International**	Jill Sheffield, M.A.	President Emeritus and Senior Advisor	USA
**Family Care International**	Ann Starrs, M.P.A.	Executive Vice President	USA

Organization/Affiliation	Name	Title	Country
**First Candle**	Marian Sokol, Ph.D., M.P.H.	President	USA
**Global Health Council**	Nils Daulaire, M.D., M.P.H.	Former President and CEO	USA
**International Labour Organization (ILO)**	Naomi Cassirer, Ph.D.	Technical Specialist with the Conditions of Work and Employment Program	Switzerland
**International Stillbirth Alliance**<br><br>**Centre for Clinical Studies, the Mater Mother's Hospital**	Vicki Flenady, M.Med.Sc.	Conference Chair<br><br>Perinatal Researcher	Australia
**Iran University of Medical Sciences**	Simin Taavoni, M.Sc.	Senior Lecturer and Midwife	Iran
**March of Dimes**	Jennifer Howse, Ph.D.	President	USA
**March of Dimes**	Chris Howson, Ph.D.	Vice President Global Programs	USA
**March of Dimes**	Doug Staples	Senior Vice President of Marketing and Communications	USA
**March of Dimes**	Marina Weiss, Ph.D.	Senior Vice President of Public Policy and Government Affairs	USA
**McGill University of Medicine, Departments of Pediatrics and Epidemiology and Biostatistics**<br><br>**GAPPS**	Michael Kramer, M.D.	James McGill Professor<br><br>SAC Member	Canada
**Norwegian Agency for Development Cooperation (NORAD)**	Helga Fogstad, M.H.A.	Coordinator, MNCH, Global Health and AIDS Department	Norway
**Obafemi Awolowo University Department of Obstetrics, Gynecology and Perinatology**	Femi Kuti, M.B.B.S, F.R.C.O.G.	Researcher	Nigeria
**PATH**	Scott Jackson, M.B.A., C.F.R.E.	Senior Vice President of External Relations	USA

Organization/Affiliation	Name	Title	Country
**PATH**	Rachel Wilson, M.P.H.	Director of Policy and Advocacy	USA
**Partnership for Maternal, Newborn and Child Health**	Andres de Francisco, M.D., Ph.D., M.Sc., D.T.M.&H.	Special Adviser, Strategy and Scientific Policy	Switzerland
**Peking University**<br><br>**GAPPS, Scientific Advisory Council**	Pang Ruyan, M.D., M.P.H.	Visiting Professor of Public Health<br><br>SAC Member	China
**Pregnancy & Perinatology Branch, National Institute of Child Health & Human Development (NICHD), NIH**	Uma Reddy, M.D., M.P.H.	Extramural Program Staff	USA
**Pregnancy & Perinatology Branch, National Institute of Child Health & Human Development (NICHD), NIH**	Catherine Spong, M.D.	Branch Manager	USA
**Save the Children, Bolivia**	Bertha Pooley, M.P.H.	National Advisor	Bolivia
**Save the Children, Saving Newborn Lives**	O. Massee Bateman, M.D., D.T.M&H.	Director	USA
**Save the Children, Saving Newborn Lives**<br><br>**GAPPS, Core Investigator**	Joy Lawn, BMedSci, M.B. B.S., M.R.C.P. (Paeds), M.P.H.	Senior Research and Policy Adviser<br><br>Core Investigator	South Africa
**Syracuse University, Maxwell School of Citizenship and Public Affairs**	Jeremy Shiffman, M.A., Ph.D.	Associate Professor of Public Administration	USA
**UNFPA**	Luc de Bernis, M.D.	Senior Advisor for Maternal Health in Africa	Kenya
**UNFPA**	Katja Iversen	Media Officer	USA
**UNICEF**	Brian Hansford	Communications Specialist	USA

Organization/Affiliation	Name	Title	Country
**UNICEF**	Mark Young, M.D., M.H.Sc.	Senior Health Advisor	USA
**US Congress**	Rep. Peter King, J.D.	Representative (R-NY)	USA
**US Congress**	Sen. Frank Lautenberg	Senator (D-NJ)	USA
**US Congress**	Erin Fogarty	Mother of a stillborn; assisting with the SUID Bill	USA
**World Bank, Human Development Network**	Joy Phumaphi	Vice President and Network Head	USA
**World Health Organization, Department of Making Pregnancy Safer**	Marie-Agnès Heine	Communications Officer	Switzerland
**World Health Organization, Department of Making Pregnancy Safer**	Monir Islam, M.D., M.P.H.	Director	Switzerland
**White Ribbon Alliance for Safe Motherhood**	Theresa Shaver, M.P.H., R.N.M.	Director	USA
